# Melanomas with activating *RAF1* fusions: clinical, histopathologic, and molecular profiles

**DOI:** 10.1038/s41379-020-0510-7

**Published:** 2020-03-02

**Authors:** Erik A. Williams, Nikunj Shah, Meagan Montesion, Radwa Sharaf, Dean C. Pavlick, Ethan S. Sokol, Brian M. Alexander, Jeff M. Venstrom, Julia A. Elvin, Jeffrey S. Ross, Julie Y. Tse, Mark C. Mochel

**Affiliations:** 1 Foundation Medicine, Inc., 150 Second Street, Cambridge, MA 02141 USA; 2 Department of Pathology, State University of New York Upstate Medical University, 766 Irving Avenue, Syracuse, NY 13210 USA; 3 Department of Pathology & Laboratory Medicine, Tufts University School of Medicine, 145 Harrison Ave, Boston, MA 02111 USA; 4 Departments of Pathology and Dermatology, Virginia Commonwealth University School of Medicine, 1200 East Marshall Street, Richmond, VA 23298 USA

**Keywords:** Melanoma, Cancer genomics

## Abstract

A subset of melanomas is characterized by fusions involving genes that encode kinases. Melanomas with *RAF1* fusions have been rarely reported, mostly in clinical literature. To investigate this distinctive group of melanomas, we searched for melanomas with activating structural variants in *RAF1*, utilizing our case archive of clinical samples with comprehensive genomic profiling (CGP) by a hybrid capture-based DNA sequencing platform. Clinical data, pathology reports, and histopathology were reviewed for each case. *RAF1* breakpoints, fusion partners, and co-occurring genetic alterations were characterized. From a cohort of 7119 melanomas, 40 cases (0.6%) featured fusions that created activating structural variants in *RAF1*. Cases with activating *RAF1* fusions had median age of 62 years, were 58% male, and consisted of 9 primary tumors and 31 metastases. Thirty-nine cases were cutaneous primary, while one case was mucosal (anal) primary. Primary cutaneous melanomas showed variable architectures, including wedge-shaped and nodular growth patterns. Cytomorphology was predominantly epithelioid, with only one case, a desmoplastic melanoma, consisting predominantly of spindle cells. *RAF1* 5′ rearrangement partners were predominantly intrachromosomal (*n* = 18), and recurrent partners included *MAP4* (*n* = 3), *CTNNA1* (*n* = 2), *LRCH3* (*n* = 2), *GOLGA4* (*n* = 2), *CTDSPL* (*n* = 2), and *PRKAR2A* (*n* = 2), all 5′ of the region encoding the kinase domain. *RAF1* breakpoints occurred in intron 7 (*n* = 32), intron 9 (*n* = 4), intron 5 (*n* = 2), and intron 6 (*n* = 2). Ninety-eight percent (*n* = 39) were wild type for *BRAF*, *NRAS*, and *NF1* genomic alterations (triple wild type). Activating *RAF1* fusions were present in 2.1% of triple wild-type melanomas overall (39/1882). In melanomas with activating *RAF1* fusions, frequently mutated genes included *TERTp* (62%), *CDKN2A* (60%), *TP53* (13%), *ARID2* (10%), and *PTEN* (10%). Activating *RAF1* fusions characterize a significant subset of triple wild-type melanoma (2.1%) with frequent accompanying mutations in *TERTp* and *CDKN2A*. CGP of melanomas may improve tumor classification and inform potential therapeutic options, such as consideration of specific kinase inhibitors.

## Introduction

The majority of melanomas harbor point mutations of *BRAF*, *NRAS*, *KIT*, or *NF1* that drive tumor growth [1, 2]. Kinase rearrangements, although less common, represent the oncogenic drivers in emerging subgroups of melanoma, often through activation of MAP kinase pathways. Rearrangements in several genes, including *BRAF*, *RET*, *ROS1*, *ALK*, *NTRK1*, and *NTRK3*, have been characterized in subsets of melanoma [2–5]. Surprisingly, despite the central role of RAF1 in the MAP kinase pathway, there are only isolated reports of *RAF1* (*CRAF*) fusions in melanomas, and the histopathologic characterizations of these tumors have been limited [6–8].

The literature on melanoma with rearrangements in kinase genes other than *RAF1* is extensive. In particular, many of these rearrangements are associated with melanomas that demonstrate characteristic spitzoid cytomorphology, leading to their classification as Spitz melanomas [9–11]. As specified in the most recent World Health Organization (WHO) classification [12], the term “Spitz melanoma” refers specifically to melanoma with both histologic changes reminiscent of Spitz nevi and a known oncogenic fusion driver, particularly of kinase-encoding genes. In contrast, “spitzoid melanoma” refers to melanoma with some morphologic resemblance to Spitz nevus, but without a known fusion driver.

Retrospective studies of spitzoid neoplasms have found activating fusions involving *BRAF, RET*, *ROS1*, *ALK*, or *NTRK1* in 39% of melanomas with spitzoid morphology and just over 50% of Spitz nevi and atypical Spitz tumors [11, 13]. In the largest reported series to date, patients with fusion-positive Spitz melanomas showed a broad age distribution, ranging from 6 to 73 years old, with a median age of 31 years [11]. While Spitz nevi and atypical Spitz tumors with *NTRK3* [14] and *NTRK1* [15, 16] fusions, as well as pigmented spindle cell nevi of Reed with *NTRK3* fusions [17], have shown distinctive clinical and histopathologic profiles, Spitz melanomas with these alterations generally do not show similarly distinguishing characteristics. Small series have characterized adult cutaneous melanomas with *NTRK* fusions, correlated with large epithelioid and amelanotic cytomorphology, and *BRAF* fusions, which have been variably correlated with spitzoid morphology [3, 4, 18, 19]. A recent study described pediatric Spitz melanomas with *MAP3K8* fusions or truncations which tended to show expansile growth, hypercellularity, deep mitoses, and ulceration [20].

Following the identification in our archive of an activating *RAF1*-fusion melanoma that responded to therapy with a MEK inhibitor [21], we performed a search of our archive of 276,645 clinical samples to identify melanoma cases with *RAF1* fusions that created known or likely activating structural variants in *RAF1*, defined as loss of the autoinhibitory domain but retention of the kinase domain. In this study, we present the first series of activating *RAF1*-fusion melanomas with clinical–pathologic correlation, detailed descriptions of several new fusion variants, and a thorough characterization of accompanying mutations.

## Materials and methods

### Cohort and genomic analyses

Comprehensive genomic profiling (CGP) was performed in a Clinical Laboratory Improvement Amendments certified, College of American Pathologists-accredited laboratory (Foundation Medicine, Inc., Cambridge, MA, USA). Approval for this study, including a waiver of informed consent and a HIPAA waiver of authorization, was obtained from the Western Institutional Review Board (Protocol No. 20152817). For quality assurance, the presence of diagnostic tumor tissue was confirmed on routine hematoxylin and eosin (H&E)-stained slides before DNA extraction. In brief, ≥60 ng of DNA was extracted from 40 μm sections of 7119 melanoma specimens, in formalin fixed, paraffin-embedded tissue blocks. The samples were assayed by CGP using adaptor ligation, and hybrid capture was performed for all coding exons from 287 (version 1) to 315 (version 2) cancer-related genes plus select introns from 19 (version 1) to 28 (version 2) genes frequently rearranged in cancer (Supplementary Table 1). Sequences were analyzed for all classes of genomic alterations, including short variant alterations (base substitutions, insertions, and deletions), copy number alterations (focal amplifications and homozygous deletions), and select gene fusions or rearrangements, by methods previously described [22–24]. Tumor mutational burden (TMB, mutations/Mb) was determined on 0.8–1.1 Mbp of sequenced DNA [24]. Microsatellite instability was determined on up to 114 loci [25].

### Mutational signatures

Mutational signatures were evaluated for all samples containing at least 20 nondriver somatic missense alterations. Signatures were given by analysis of the trinucleotide context and profiled using the Sanger COSMIC signatures of mutational processes in human cancer [26]. A positive signature was determined if a sample had at least a 40% fit to a mutational process [26]. The COSMIC UV signature is dominated by C > T transition mutations in a CC or TT dinucleotide setting [27].

### Clinical–pathological analysis of melanoma cohort harboring activating *RAF1* fusions

The cohort of melanomas harboring activating *RAF1* fusions comprised 40 cases, each from a different patient. Assays with CGP (Foundation Medicine Cambridge, MA, USA) occurred during clinical care at other institutions. Clinicopathological data including patient age, gender, tumor site, tumor diameter, and stage were extracted from the accompanying pathology reports.

H&E stained sections from each of the 40 cases were assessed retrospectively by two board-certified dermatopathologists (JYT and MCM). Histologic parameters assessed on primary tumors included tumor silhouette (dome shape, plaque-like growth, nodular growth, etc.), symmetry, shape of the tumor base (wedge-shaped, flat, bulbous, etc.), presence of epidermal involvement (and intraepidermal growth patterns), ulceration, maturation, deep nested growth, fascicular growth, associated dermal fibrosis, Breslow depth, mitotic rate, grade of solar elastosis [28], and tumor-infiltrating lymphocytes. Cytologic features, assessed on all cases, included predominant cytomorphology (epithelioid, spindled, mixed epithelioid and spindled), cytoplasmic color and abundance, and nuclear features of chromatin quality, nucleolar prominence, and degree of pleomorphism.

The H&E slides were independently diagnostic of melanoma for primary tumors and for pigmented metastases. In contrast, H&E slides were not independently diagnostic for cases of metastatic melanoma that showed nonpigmented malignant epithelioid proliferations. Those cases required diagnostic corroboration with accompanying pathology reports for relevant historical and immunohistochemical details (e.g., documented Melan-A and S100 positivity to confirm the diagnosis of melanoma vs. other malignant epithelioid tumors).

Quantitative data were analyzed using the Fisher exact test owing to the categorical quality of the data and the size of the cohort. For TMB comparison between two groups, the nonparametric Mann–Whitney *U* test was used. A two‐tailed *P* value of <0.05 was considered to be statistically significant.

## Results

### Clinical–pathologic features

From an internal series of 7119 melanomas that had undergone prior hybrid capture-based DNA sequencing, 40 cases (0.6%), each from a different patient, featured gene rearrangements that created known or likely activating structural variants in *RAF1*, defined as loss of the autoinhibitory domain but retention of the kinase domain.

Among patients with activating *RAF1*-rearranged melanomas, the ages ranged from 34 to 86 years, with a median of 62 years. There were 23 males and 17 females. All patients had clinically advanced disease. Clinical staging ranged from at least stage 2A to stage 4, with the majority of cases documented at stage 4 (*n* = 25 of 40; 63%) and most of the remaining cases at either stage 3A or 3B (*n* = 10 of 40; 25%). Sequencing was performed on the original primary tumor in 8 primary cutaneous melanomas and on 31 metastatic disease samples. Of the metastatic samples, sites included regional lymph nodes (*n* = 8), in-transit metastasis (*n* = 1), and distant lymph nodes (*n* = 3). Additional distant metastatic sites included skin (subcutaneous (*n* = 4) and dermal (*n* = 2)), soft tissue (*n* = 3), brain (*n* = 2), lung (*n* = 2), and one each involving liver, omentum, small intestine, adrenal, bone, and spleen. Thirty-nine cases were consistent with either primary cutaneous melanoma or metastatic melanoma from a skin primary, while one case was a primary melanoma of anal mucosa.

Primary cutaneous tumors occurred on the extremities and trunk showed a mean tumor diameter of 16 mm (range 3–40 mm), mean thickness of 6.6 mm (range 2.3–17 mm), and mean mitotic rate of 5.5 per mm^2^ (range 1–14 per mm^2^) (Table 1). Melanomas were classified as nodular (3), superficial spreading (2), unclassified (2), and desmoplastic (1). The two melanomas with an unclassified subtype comprised one specimen of broadly ulcerated and deeply invasive melanoma without assessable lesional edges (case 1) and another specimen of residual melanoma deep to scar (case 4).

**Table 1:** Clinical and pathologic features of primary *RAF1*-fusion cutaneous melanomas.

Case No.	Gender	Age (years)	Location	Diameter (mm)	Type	Cytology	Thickness (mm)	Ulcer	TMR (#/mm sq.)
1	Male	52	Leg	40	Unclassified	Epithelioid	17.0	Present	8
2	Female	69	Leg	9	Nodular	Epithelioid	2.8	Absent	3
3	Female	54	Back	13	SSM	Epithelioid	6.3	Absent	4
4	Female	39	Abdomen	9	Unclassified	Epithelioid	5.0	Absent	14
5	Female	74	Arm	23	Desmoplastic	Spindled	13.0	Absent	2
6	Female	65	Back	15	SSM	Epithelioid	3.3	Absent	10
7	Female	34	Leg	3	Nodular	Epithelioid	3.0	Absent	1
8	Male	68	Abdomen	19	Nodular	Epithelioid	2.3	Absent	2

*TMR* tumor mitotic rate, # number, *SSM* superficial spreading melanoma.

**Fig. 1:**
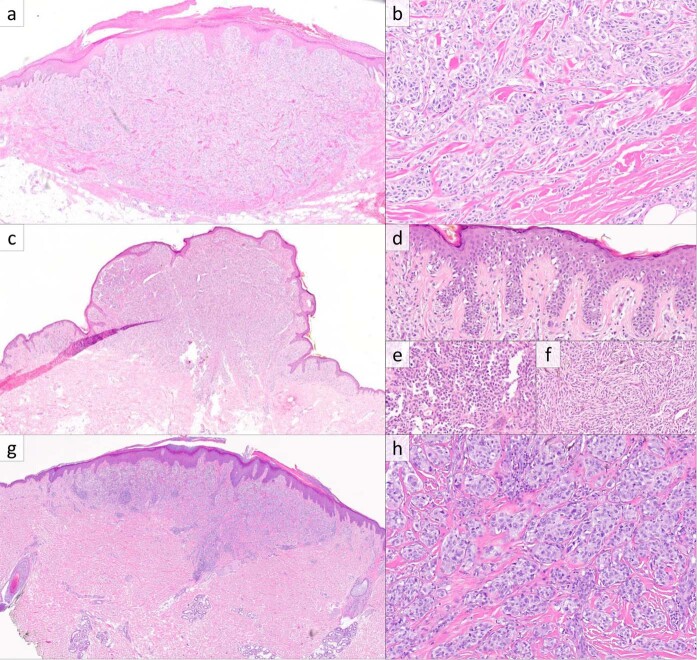
*RAF1*-fusion melanoma, primary cutaneous lesions. **a** Histopathologic examination of case 2 reveals a melanocytic neoplasm with a slightly domed surface and wedge-shaped base centered in the dermis (H&E, ×20). **b** The deep aspect of case 2 consists of nested large epithelioid melanocytes with associated dense collagen fibers and scattered mitotic figures (H&E, ×200). **c** Case 3 showed an exophytic component with a plaque-like growth pattern at the periphery (H&E, ×20). For case 3, the radial growth phase at the periphery showed intraepidermal growth with pagetoid scatter (**d**), while the vertical growth phase contained predominantly epithelioid melanocytes (**e**) with a deep zone of fascicular growth of spindled cells with focal cytoplasmic pigmentation (**f**) (H&E, ×200, ×400, and ×400). **g**, **h** Case 8 showed a melanocytic neoplasm with a domed surface and wedge-shaped base with nested growth in the deep aspect with dense collagen fibers and scattered mitoses (H&E, ×20 and ×200).

Histopathologic examination of the eight primary cutaneous melanomas revealed heterogeneous features (Fig. 1). Three cases showed domed surfaces with wedge-shaped bases (Fig. 1a–g), two showed nodular growth in the dermis and subcutis, one showed nodular and diffusely infiltrative growth in the dermis and subcutis, one appeared plaque like with an exophytic component (Fig. 1c), and the desmoplastic melanoma appeared as a haphazard spindle cell proliferation in the dermis and subcutis with fibrosis and lymphoid aggregates. Four cases were plainly asymmetric, while four were subtly asymmetric. Four cases showed epidermal involvement, with two demonstrating melanoma in situ extending beyond the dermal proliferation (corresponding to the radial growth phases) and the remaining two showing focal involvement overlying the dermal component. Of those with epidermal involvement, all four showed some degree of epidermal hyperplasia, three displayed pagetoid growth, and a single case showed confluent growth with epidermal effacement.

Regarding the dermal component of the primary cutaneous melanomas, significant maturation with depth was not seen in any case. The presence of deep nested growth was seen in four cases, including all three with wedge-shaped arrangement (Fig. 1b–h). Three cases were associated with densely eosinophilic and slightly thickened collagen fibers, while one case showed a minute focus of fibrosis with increased fibroblasts and pale myxoid stroma. All cases contained mitotic figures within their deep aspects. Solar elastosis tended to be scant; by the WHO scoring system [28], two had grade 0 solar elastosis, four had grade 1, one had grade 2, and one could not be determined owing to lack of tumor-free and evaluable dermis in the H&E sections. Tumor-infiltrating lymphocytes were absent or sparse in three cases, nonbrisk in four cases, and brisk in one case.

Among the eight cutaneous melanomas, melanocytic cytology was predominantly epithelioid in seven cases, and spindled in the desmoplastic melanoma case. Among the seven predominantly epithelioid cases, one showed focally spindled growth in the vertical growth phase (Fig. 1f). Their cytoplasm tended to be amphophilic (four cases) to palely eosinophilic (three cases), while one case had densely eosinophilic cytoplasm. Cytoplasmic quantity in the epithelioid cases was moderate to abundant in five and relatively scant in two. Only two showed cytoplasmic pigmentation (Fig. 1f), which was focal in both cases. Nuclear size was medium to large, and chromatin was heterogeneous (admixed dense and pale) in all cases. Nucleoli were prominent in three cases, small and distinct in two cases, and indistinct in three cases. Nuclear pleomorphism was judged to be severe in four cases and mild to moderate in the other four. No cases showed convincing Spitz-nevus-like cytomorphology (i.e., voluminous, homogeneous cytoplasm with sharp borders) or pulverocytic cytology characterized by pale, finely pigmented cytoplasm.

**Fig. 2:**
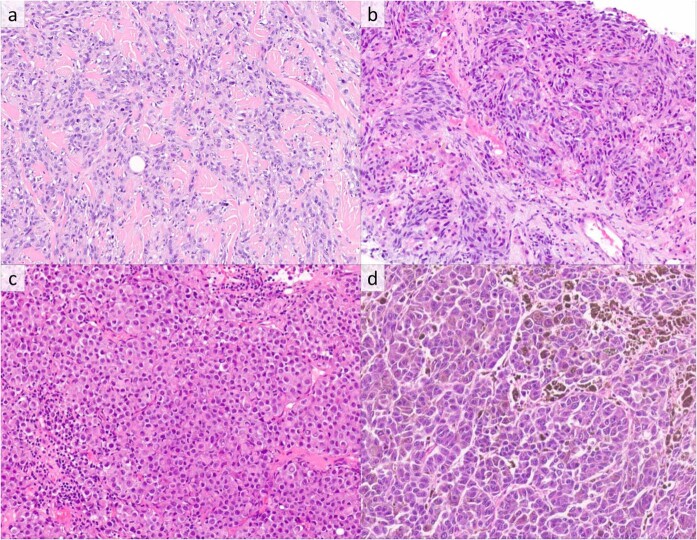
*RAF1*-fusion melanoma, metastatic lesions. **a** Metastatic melanoma involving soft tissue with epithelioid cytomorphology (H&E, ×400). **b** Metastatic melanoma involving liver with admixed epithelioid and spindled morphology, a pattern seen in only two metastases (H&E, ×400). **c** Metastatic melanoma involving a lymph node with eccentric dense eosinophilic cytoplasm, imparting a rhabdoid cytomorphology (H&E, ×400). **d** Metastatic melanoma involving small intestine with large epithelioid cytology with cytoplasmic pigmentation (H&E, ×400).

Among the 31 nodal and distant melanoma metastases, histopathology showed variable combinations of diffuse and nodular growth. While all metastatic tumors showed predominantly epithelioid cytomorphology (Fig. 2a), six (19%) had eccentric dense eosinophilic cytoplasm paired with large nuclei with prominent nucleoli, imparting a rhabdoid appearance (Fig. 2c), and two (6%) showed mixed epithelioid and spindled cytology (Fig. 2b). Cytoplasm was amphophilic (15 cases), palely eosinophilic (8 cases), and densely eosinophilic (8 cases). Only five metastatic cases showed cytoplasmic pigmentation, which was focal in three cases and diffuse in two (Fig. 2d). Nuclear size was medium to large in all cases, and nucleoli were prominent in 13 (42%). Nuclear pleomorphism was severe in 16 cases (52%) and mild-moderate in 15 cases (48%).

### Comprehensive genomic profiling

**Fig. 3:**
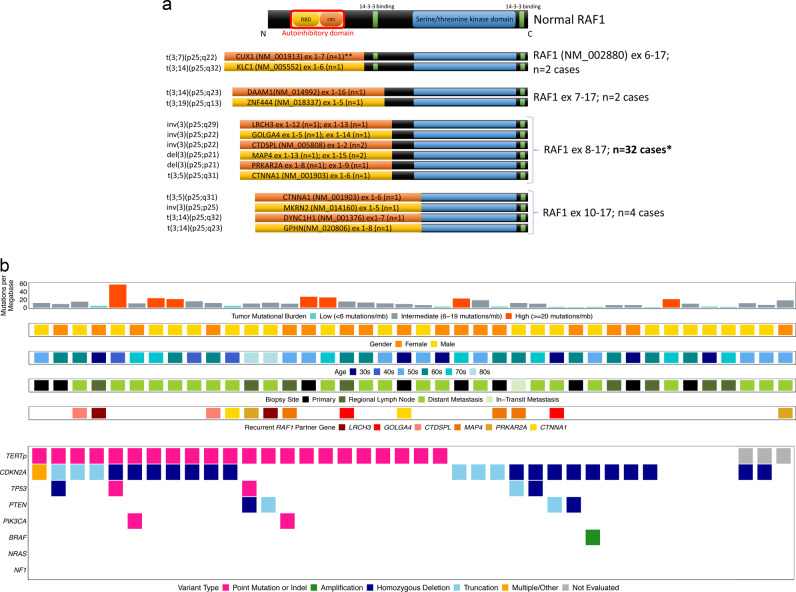
Molecular profiles of activating *RAF1*-fusion melanomas. **a** Schematic of functional domains of RAF1 and structure of activating RAF1 fusions showing loss of the autoinhibitory domain but retention of the RAF1 kinase domain. Recurrent *RAF1* breakpoints were identified in introns 5–7 and 9, i.e., 5′ of the region encoding the kinase domain. **b** Summary of clinical features and molecular alterations in activating *RAF1-*fusion melanomas. RBD Ras-binding domain, CRD cysteine-rich domain, *TERTp* *TERT* promoter. *Of these 32 cases, only recurrent partners with RAF1 are depicted. **RAF1 partner exon size is not representative in any case.

5′ rearrangement partners were predominantly intrachromosomal (*n* = 17), and recurrent partners included *MAP4* (*n* = 3), *CTNNA1* (*n* = 2), *LRCH3* (*n* = 2), *GOLGA4* (*n* = 2), *CTDSPL* (*n* = 2), and *PRKAR2A* (*n* = 2) (Fig. 3a). *RAF1* breakpoints occurred in intron 7 (*n* = 32), intron 9 (*n* = 4), intron 5 (*n* = 2), and intron 6 (*n* = 2), i.e., all 5′ of the region encoding the kinase domain (Fig. 3a).

Of the activating *RAF1*-fusion melanomas, 98% (*n* = 39) were wild type for *BRAF*, *NRAS*, and *NF1* genomic alterations (triple wild type) vs. 26% (*n* = 1843/7079) of the melanoma cohort without *RAF1* fusion (*p* < 0.0001, Fisher’s exact test). The most frequently mutated other genes in *RAF1*-fusion melanomas were *TERTp* (62%; 23/37), *CDKN2A* (60%; 24/40), *TP53* (13%; 5/40), *SPTA* (12%; 3/26), *FOXP1* (12%; 4/27), *ARID2* (10%; 3/29), and *PTEN* (10%; 4/40) (Fig. 3b). Median TMB was 10.2 mut/Mb (range 0–57.4, 35% 10–20, 18% >20), similar to the primary skin and anus melanoma cohort without activating *RAF1* fusions (median TMB = 13.8, 19% 10–20, 36% >20; *p* = 0.1, Mann–Whitney *U* test). Copy number plots showed complex gains and losses. All evaluated cases were microsatellite stable. Of the 28 cases with the requisite number of mutations for UV signature identification, 22 harbored a positive UV signature (79%). Of the remaining six cases, three had a UV signature <40% (10, 29, and 38%).

Comparison of tumors sequenced from the primary sites, regional metastases, and distant metastases was performed. Tumors sequenced from primary sites vs. metastases showed similar percentages of genomic alterations, including in *TERTp*, *CDKN2A*, *TP53*, and *PTEN* (56% vs. 64%, 67% vs. 58%, 11% vs. 13%, and 11% vs. 10%, respectively). Similarly, no significant differences in percentages of genomic alterations were identified between tumors sequenced from regional vs. distant metastases.

Three additional cases with *RAF1* rearrangements that did not result in loss of the regulatory domain of *RAF1* were identified, including two *RAF1* intergenic rearrangements (exon 2–6 inversion and exon 6–7 duplication) and one *OPRM1–RAF1* rearrangement with *RAF1* breakpoint at exon 2. The exon inversion case was of vulvar origin with two copy loss of *CDKN2A*, while the remaining two cases were cutaneous in origin and had pathogenic *BRAF* mutations.

## Discussion

To our knowledge, this study represents the first series of activating *RAF1*-fusion melanomas with clinicopathologic correlation and detailed characterization of genetic alterations. Activating *RAF1* fusions represent a significant subset of triple wild-type melanoma (2.1% of all triple wild-type melanoma). Recurrent fusion partners and recurrent *RAF1* breakpoints were present. Frequent accompanying mutations in *TERTp* and *CDKN2A* were identified, typical for skin primary melanoma, and TMB was not significantly different from primary skin and anus melanoma cases without activating *RAF1* fusions.

Reports of activating *RAF1* fusions in melanocytic neoplasms have been rare. In one study seeking therapeutically targetable gene fusions in multiple cancer types, FISH for *BRAF* and *RAF1* performed on 131 melanomas identified one *BRAF* rearrangement and one *RAF1* rearrangement [6]. A more recent study of kinase fusions across large numbers of various malignancies found four cases of *RAF1* fusion out of 397 melanomas [29]. A whole-genome study of 183 melanomas found *RAF1* fusions in two melanomas (with partners *CDH3* and *GOLGA4*): one triple wild type and one with an *NF1* comutation [30]. In a study of 21 large to giant congenital nevi, one case was found to have a *SOX5–RAF1* fusion [31]. This congenital nevus, like that of an *ALK*-fused nevus in the same study, lacked comutations of *NRAS* and *BRAF*. A striking case report also showed a fusion of *SASS6–RAF1* in a giant congenital nevus that gave rise to melanoma with rhabdomyosarcomatous differentiation [32]. While a balanced translocation was found in the background congenital nevus, an unbalanced translocation was noted in the rhabdomyosarcomatous component.

Of note, activating *RAF1* fusions are also found in a small proportion of thyroid carcinomas, prostatic adenocarcinomas, and pilocytic astrocytomas [29, 33, 34]. Atefi et al. described *RAF1* missense point mutation R391W in one melanoma cell line that lacked common driver mutations and showed resistance to vemurafenib despite MAPK signaling [7]. One report described a melanoma with *GOLGA4* fused to exons 8–17 of *RAF1*, retaining the *RAF1* kinase domains, and with accompanying mutations in *CTNNB1* and *CDKN2A* [8]. Importantly, this patient’s melanoma showed a marked clinical response to therapeutic MEK inhibition, as indicated by serial PET scans.

Prior histopathologic descriptions of activating *RAF1*-fusion melanomas are limited. We observed various architectural patterns, including wedge-shaped growth with associated epidermal hyperplasia and deep nested melanocytes in three cases, reminiscent of some Spitz tumors with *ALK* and *NTRK1* fusions [10]. In contrast to Spitz tumors with fusions of *ALK*, *NTRK1*, and *ROS1*, which are usually compound with a prominent epidermal component, epidermal involvement in the *RAF1*-fused melanomas was typically limited or absent [9, 10, 16, 35, 36]. Characteristic spitzoid cytomorphology was not observed. Rather, we noted a somewhat heterogeneous group of tumors with predominantly epithelioid melanocytic cytomorphology with amphophilic to eosinophilic cytoplasm and medium to large nuclei with heterogeneous chromatin and often prominent nucleoli. In some cases, the large epithelioid cytology resembled that reported in *NTRK*-fusion melanomas, as well as in some Spitz tumors with *ALK* translocation [4, 13, 36]. In our cases of primary cutaneous melanoma with activating *RAF1* fusions, a distinctive growth pattern, such as the fascicular pattern of *ALK1*-fused Spitz tumors, was not seen. Nevertheless, typical histopathologic features of melanoma, including asymmetry, lack of maturation, cytologic atypia, and dermal mitotic activity, were readily identifiable in the cases evaluated in our study.

Triple wild-type melanomas (*BRAF*, *RAS*, and *NF1* wild type) typically lack a UV signature [37]. In our series, however, activating *RAF1*-fusion melanoma was almost entirely triple wild type, cutaneous, and UV driven. Given the frequent concurrent mutations in *TERTp*, *CDKN2A*, and *TP53*, and the relatively low TMB (median = 10.2 mut/Mb), these tumors may fit best into the low cumulative sun damage group, as described in the current WHO classification [28]. Concordant with this classification, primary cases in our cohort generally lacked significant solar elastosis.

The *RAF1* fusions we identified appear to be pathogenic, given that the most significant regulatory mechanism for RAF1 is the direct association of the N-terminal autoinhibitory domains to the kinase domain. Loss of this domain but retention of the kinase domains, as seen in each of our cases (Fig. 3a), would cause autonomous, unregulated activation of kinase activity [38].

Limitations of this study include its retrospective nature and the distinct population of patients highly enriched for aggressive tumors, mostly metastatic to distant sites. Typically, extensive genomic testing is performed on advanced malignancies from patients whose oncologists are seeking targeted therapies. Thus, this patient population may not be representative of the general population of patients with melanoma. We note that the median age of 62 years for *RAF1*-fusion melanomas in this study is significantly older than that reported in prior melanocytic tumors with gene fusions, which tend toward pediatric and young adult patients. While an actual age difference may exist, this age discrepancy may be attributable to disparate study cohorts: our cohort was selected from melanomas with proven aggressive behavior, while other fusion-positive melanocytic tumor cohorts often were selected for Spitz morphology, often in diagnostically challenging cases, and therefore enriched for young patients. Finally, while our review of histologic slides from all cases enabled us to confirm histopathologic diagnoses, particularly for cases of primary cutaneous melanoma and pigmented metastatic lesions where H&E slides were independently diagnostic, some cases required corroboration with details from the accompanying pathology reports (e.g., for metastatic melanomas with histologic slides showing malignant epithelioid proliferations, we relied on corresponding pathology reports for confirmatory immunohistochemical details).

CGP of melanomas may provide insights into pathogenesis, as well as potential therapeutic options. Additional studies will be needed to correlate the finding of activating *RAF1* fusions in melanoma with prognostic data and treatment outcomes. Prognostic data will also enable the comparison of *RAF1*-fused melanomas by comutations, such as *TERTp*, which has been shown to be an important prognostic marker for spitzoid melanocytic neoplasms [39]. Furthermore, the spectrum of melanocytic lesions with activating *RAF1* rearrangements may be wider than the highly selected, aggressive tumors examined in this study. Overall, our findings provide a compelling rationale for consideration of CGP of melanomas, which may offer insights into melanoma biology and potentially inform therapeutic options, including specific kinase inhibitors.

## Supplementary information

Supplementary Table 1
